# Denervation as a Common Mechanism Underlying Different Pulmonary Vein Isolation Strategies for Paroxysmal Atrial Fibrillation: Evidenced by Heart Rate Variability after Ablation

**DOI:** 10.1155/2013/569564

**Published:** 2013-08-24

**Authors:** Kejing Wang, Dong Chang, Zhenliang Chu, Yanzong Yang, Lianjun Gao, Shulong Zhang, Yunlong Xia, Yingxue Dong, Xiaomeng Yin, Peixin Cong, Jingjing Jia

**Affiliations:** ^1^Department of Cardiology, First Affiliated Hospital of Dalian Medical University, Dalian 116011, China; ^2^Department of Cardiology, Jiaxing Second Hospital, Jiaxing 314000, China

## Abstract

*Backgrounds*. Segmental and circumferential pulmonary vein isolations (SPVI and CPVI) have been demonstrated to be effective therapies for paroxysmal atrial fibrillation (PAF). PVI is well established as the endpoint of different ablation techniques, whereas it may not completely account for the long-term success. *Methods*. 181 drug-refractory symptomatic PAF patients were referred for segmental or circumferential PVI (SPVI = 67; CPVI = 114). Heart rate variability (HRV) was assessed before and after the final ablation. *Results*. After following up for 62.23 ± 12.75 months, patients underwent 1.41 ± 0.68 procedures in average, and the success rates in SPVI and CPVI groups were comparable. 119 patients were free from AF recurrence (SPVI-S, *n* = 43; CPVI-S, *n* = 76). 56 patients had recurrent episodes (SPVI-R, *n* = 21; CPVI-R, *n* = 35). Either ablation technique decreased HRV significantly. Postablation SDNN and rMSSD were significantly lower in SPVI-S and CPVI-S subgroups than in SPVI-R and CPVI-R subgroups (SPVI-S versus SPVI-R: SDNN 91.8 ± 32.6 versus 111.5 ± 36.2 ms, rMSSD 47.4 ± 32.3 versus 55.2 ± 35.2 ms; CPVI-S versus CPVI-R: SDNN 83.0 ± 35.6 versus 101.0 ± 40.7 ms, rMSSD 41.1 ± 22.9 versus 59.2 ± 44.8 ms; all *P* < 0.05). Attenuation of SDNN and rMSSD remained for 12 months in SPVI-S and CPVI-S subgroups, whereas it recovered earlier in SPVI-R and CPVI-R subgroups. Multivariate logistic regression analysis identified SDNN as the only predictor of long-term success. *Conclusions*. Beyond PVI, denervation may be a common mechanism underlying different ablation strategies for PAF.

## 1. Introduction

Segmental pulmonary vein isolation (SPVI) and circumferential pulmonary vein isolation (CPVI) are two established therapeutic techniques for catheter ablation of paroxysmal atrial fibrillation (PAF) [1]. SPVI is believed to eliminate the initiating triggers of AF, whereas CPVI aims to electrically isolate the pulmonary veins and in doing so probably affects both the trigger and substrate. Pulmonary vein isolation (PVI) has been widely accepted as the endpoint of both strategies and is regarded as the cornerstone of PAF ablation. However, recurrence of AF remains problematic after PVI. PVI may not completely account for the long-term efficiency of ablation.

Among the complex and disputed mechanisms of AF, the cardiac autonomic nervous system (ANS) may play a crucial role in its initiation and maintenance [2, 3]. Furthermore, accumulating evidence indicates that both SPVI and CPVI induce denervation [4], which may increase the success rate of ablation [5–8]. Denervation may therefore be a common mechanism underlying the therapeutic effects of different procedures for treating PAF. However, the effect of denervation on the long-term efficiency of AF ablation has not been well clarified.

The purpose of the present study was 2-fold. First, we determined the effect of different ablation procedures on autonomic function and evaluated the effect of denervation on the long-term success rate of AF ablation. Second, we elucidated whether denervation is a common mechanism underlying different ablation strategies beyond PVI.

## 2. Methods

### 2.1. Study Population

One hundred eighty-one consecutive patients with drug-refractory symptomatic PAF between March 2005 and July 2007 were included in the present study. Most patients had once received more than one antiarrhythmic agent. 138 had received amiodarone (76.2%), 108 patients were treated with propafenone (59.7%), and 98 with *β*-receptor blocker (54.1%). The patients underwent SPVI (*n* = 67) or CPVI (*n* = 114) after confirmation of the absence of intracardiac thrombi by transesophageal echocardiography. Exclusion criteria were conditions that may interfere with the analysis of cardiac autonomic function indicated by heart rate variability (HRV) parameters, including sick sinus syndrome, pacemaker implantation, diabetes mellitus, thyroid dysfunction, pacing rhythm, and *β*-blocker therapy. Antiarrhythmic agents were discontinued for at least 5 half-lives before the study. In the periprocedural period, low-molecular-weight heparin was administered subcutaneously.

### 2.2. Electrophysiological Study and Catheter Ablation

CT scanning of the PVs and LA was performed before the procedure. All patients were given written informed consent. Local anesthesia was performed with 2% Lidocaine. Under fluoroscopic guidance (Innova 2000, GE Co., USA), two multipolar catheters (Cordis Webster Co., USA) were placed at the coronary sinus (CS) and right ventricle through the left femoral vein. Two transseptal sheaths were introduced into the right femoral vein, and after dual trans-septal puncture was performed, anticoagulation was started by a bolus administration of 5000–8000 IU heparin followed by continuous intravenous heparin infusion to maintain an activated clotting time of >250 seconds. An ablation catheter (NaviStar ThermoCool, Biosense Webster Inc.) and a mapping catheter (LASSO, Biosense Webster Inc., Diamond Bar, CA, USA) were inserted through the sheaths.

For SPVI, pulmonary vein (PV) ostia were confirmed by PV angiography. The electrical connections between the left atrium (LA) and PVs were circumferentially mapped, and irrigated ablation was applied at the PV-LA junction with the earliest PV potential. For CPVI, three-dimensional LA and PV anatomy were reconstructed by using electroanatomic mapping systems (CARTO, Biosense Webster Inc.). After identification of the PV ostia, linear ablation was performed by encircling ipsilateral PVs 0.5–1.0 cm outside the PV ostia with an irrigated catheter. A circular mapping catheter was used to identify residual conduction gaps on ablation lines. Ablation was set with a maximum temperature of 43°C and maximum power of 30–35 W. The end-points for both procedures were PVI [9, 10]. Given the trigger firing arising from superior vena cava (SVC), isolation of SVC was required. If the patients experienced atrial flutter, an additional ablation line was drawn in the mitral isthmus and/or tricuspid isthmus [1]. 

### 2.3. Holter Recordings

Serial 24-Holter recordings were obtained at baseline and 2 or 3 days after the final procedure to analyze HRV. The time-domain measures of HRV include the standard deviation of all NN intervals (SDNN), standard deviation of the average values of the NN intervals in all 5-min segments (SDANN), percentage of adjacent NN interval differences >50 ms (PNN50), and root mean square of the differences between adjacent NN intervals (rMSSD). The frequency-domain measures of the HRV included the ultralow-frequency (ULF: <0.0033 Hz) power, very-low-frequency (VLF: 0.0033–0.04 Hz) power, low-frequency (LF: 0.04–0.15 Hz) power, high-frequency (HF: 0.15–0.40 Hz) power, and the ratio of the LF to HF power (LF/HF). In the present study, aberrant signals such as AF and supraventricular or ventricular premature beats were excluded from the HRV analysis. Holter recordings with >20% AF were excluded from analysis.

### 2.4. Patient Followup

After ablation, patients received anticoagulation with warfarin for at least 3 months to maintain INR between 2 and 3. The patients were discharged without antiarrhythmic therapy. Patients were followed up for at least five years in the outpatient department or through telephone interviews. They were encouraged to report any symptoms suggestive of tachycardia and to undergo 12-lead electrocardiography (ECG) or 24-hour Holter recording every 3 to 6 months after ablation. AF recurrence was defined as atrial tachyarrhythmias sustained for more than 30 seconds that occurred 3 months after the procedure. The first 3 months were regarded as a blank period. Antiarrhythmic agents were only given to patients with recurrent AF who were experiencing significant symptoms during followup.

### 2.5. Statistical Analysis

All values are expressed as the means ± standard deviation. We analyzed HRV before and after the final ablation. The frequency-domain measurements of the HRV (TF, ULF, VLF, LF, and HF) were logarithmically transformed to normalize the distribution. Categorical variables expressed as numbers and percentages were compared using a chi-square test. Comparisons of continuous variables in different ablation groups were performed using an unpaired *t*-test (for normally distributed data) or Mann-Whitney *U* test (for nonparametric data). A paired *t*-test (for normally distributed data) or Wilcoxon signed-rank test (for nonparametric data) was used to compare HRV parameters before and after ablation in the SPVI or CPVI groups. Multivariate logistic regression analysis was performed to determine independent predictors of long-term efficiency. *P* < 0.05 was considered statistically significant. All tests were performed by SPSS 11.0 software (Chicago, IL, USA).

## 3. Results

### 3.1. Population Characteristics and Ablation Results

One hundred seventy-five patients were successfully followed up (Group SPVI = 64; Group CPVI = 111). Baseline clinical characteristics were comparable among patients undergoing SPVI or CPVI, as shown in Table 1. PVI was achieved in all patients. Following up 62.23 ± 12.75 months, each patient underwent an average of 1.41 ± 0.68 procedures; the rate of freedom from atrial tachyarrhythmia was 67.2% and 68.5% after SPVI or CPVI, respectively (*P* > 0.05). Based on the 5 year follow-up outcomes, patients who underwent SPVI or CPVI were further divided into subgroups as follows: SPVI-S group (success), SPVI-R group (recurrence), CPVI-S group (success), and CPVI-R group (recurrence).

Total ablation time was 45.4 ± 14.4 minutes for SPVI approach and 69.6 ± 25.2 minutes for the CPVI approach (*P* < 0.001), whereas success and recurrence subgroups of either technique did not differ significantly in terms of ablation time (SPVI-S 47.5 ± 14.6 minutes versus SPVI-R 40.2 ± 12.9 minutes; CPVI-S 70.1 ± 25.4 minutes versus CPVI-R 68.5 ± 25.2 minutes; *P* > 0.05). Occurrence of vagal reflexes, which was defined as sinus bradycardia (HR < 40 bpm), asystole, atrioventricular block, or hypotension (systolic blood pressure <90 mmHg) that occurred within a few seconds after radiofrequency application, was higher in the CPVI group than in the SPVI group (15.3% versus 6.3%, *P* < 0.01), whereas the occurrences of vagal reflexes were identical between success and recurrence subgroups. Abolition of all vagal reflexes was not required in the present study.

### 3.2. Changes of Heart Rate Variability

**Figure 1:**
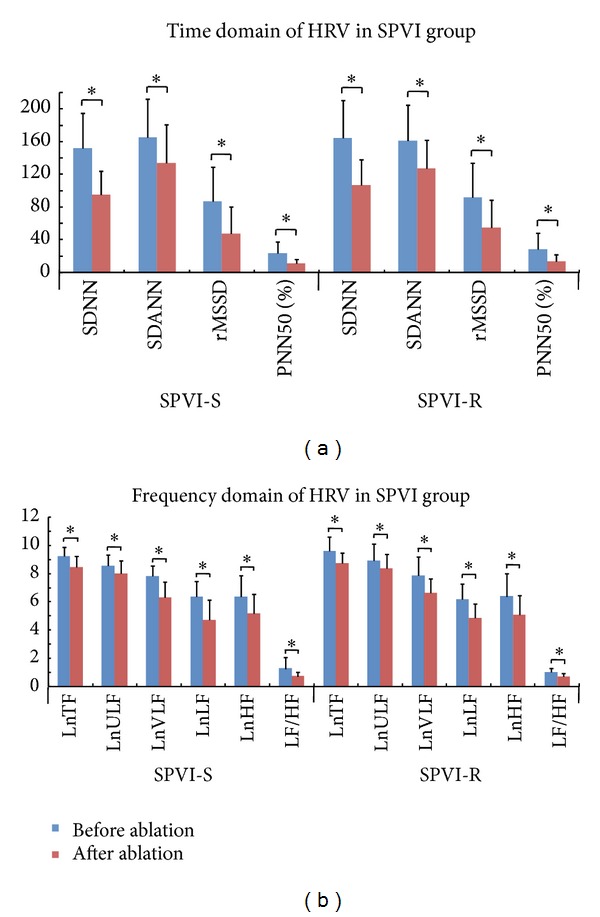
Comparison of HRV parameters before and after SPVI. (a) Time-domain parameters of HRV significantly decreased after SPVI in both the success and recurrence subgroups (*P* < 0.05 versus before ablation). (b) Frequency-domain characteristics of HRV significantly decreased in both the success and recurrence subgroups (*P* < 0.05 versus pre-ablation). HRV: heart rate variability; SPVI-S: success subgroup for segmental pulmonary vein isolation; SPVI-R: recurrence subgroup for segmental pulmonary vein isolation; CPVI-S: success subgroup for circumferential pulmonary vein isolation; CPVI-R: recurrence subgroup for circumferential pulmonary vein isolation; SDNN: standard deviation of all NN intervals (ms); SDANN, standard deviation of the averages of the NN intervals in all 5-min segments (ms); rMSSD: root mean square root of the differences between adjacent NN intervals (ms); PNN50, percentage of adjacent NN interval differences >50 ms; ULF: ultralow-frequency power; VLF: very-low-frequency power; LF, low-frequency power; HF, high-frequency power. Frequency domain parameters of HRV including TF, ULF, VLF, and LF and HF were logarithmically transformed to normalize the distribution (LnTF, LnULF, LnVLF, LnLF, and LnHF; units: ms^2^). *P < 0.05.

**Figure 2:**
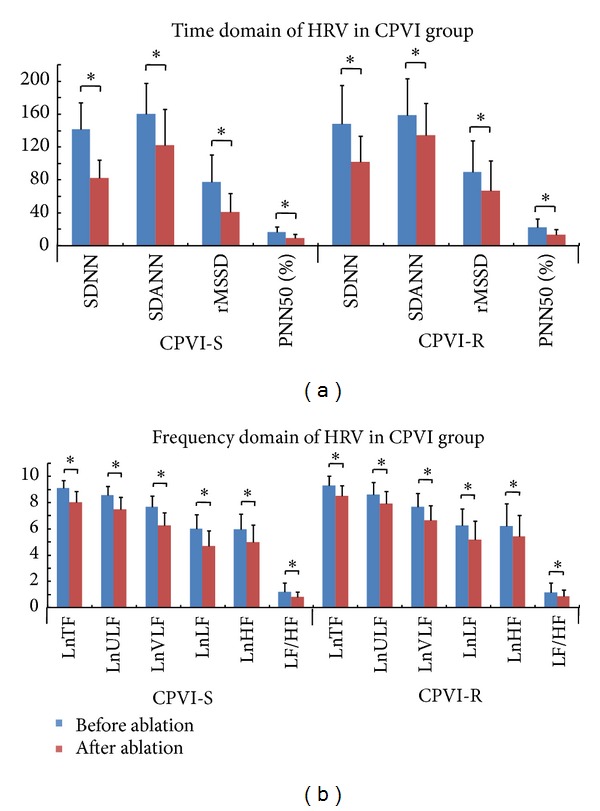
Comparison of HRV before and after CPVI. (a) Time-domain parameters of HRV significantly decreased after CPVI, both in the success and recurrence subgroups. (b) Frequency-domain characteristics of HRV significantly decreased after CPVI, both in the success and recurrence subgroups. Abbreviations are as in Figure 1. *P < 0.05.

**Figure 3:**
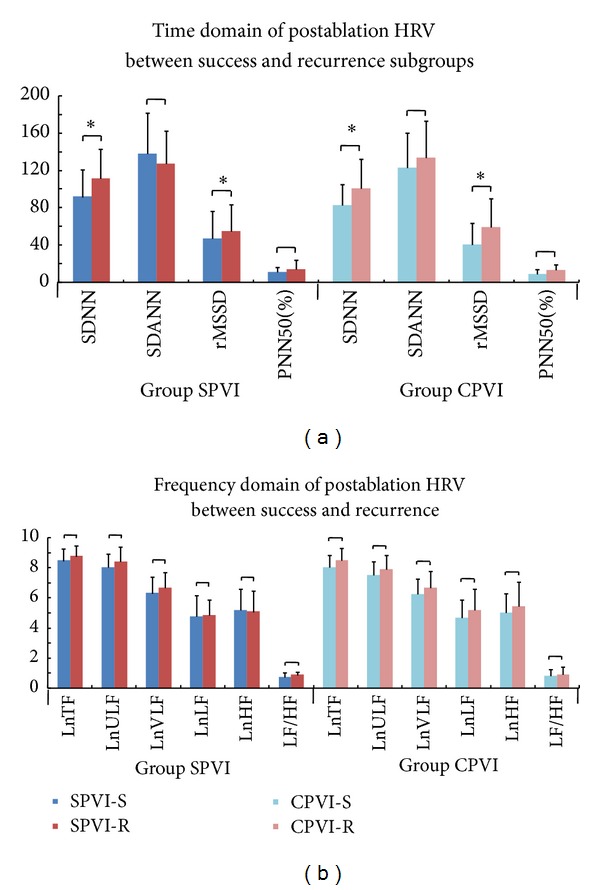
Comparison of postablation HRV between the success and recurrence subgroups. (a) After SPVI or CPVI, SDNN and rMSSD were significantly lower in the success subgroups (SPVI-S and CPVI-S) than in the recurrence subgroups (SPVI-R and CPVI-R). (b) After SPVI or CPVI, there were no significant differences in the frequency-domain parameters of HRV between subgroups (SPVI-S versus SPVI-R; CPVI-S versus CPVI-R, *P* > 0.05). Abbreviations are as in Figure 1. *P < 0.05.

**Figure 4:**
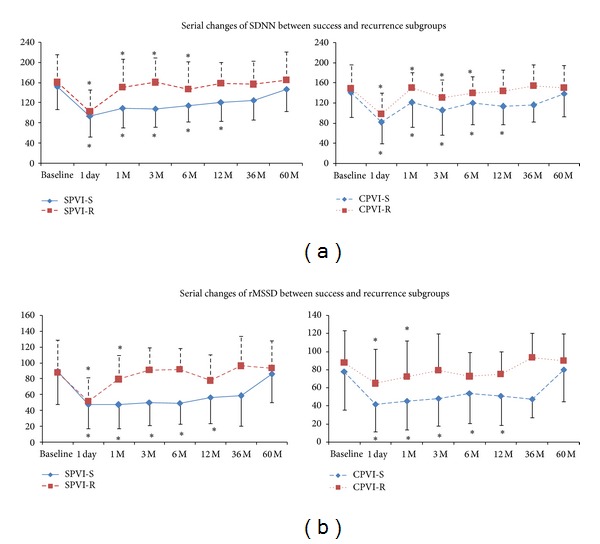
Serial changes of SDNN and rMSSD during five-year follow-up. (a) SDNN remained attenuated during 12-month follow-up in SPVI-S and CPVI-S subgroups, while recovered after 6 months in SPVI-R and CPVI-R subgroups. (b) rMSSD remained attenuated during 12-month follow-up in SPVI-S and CPVI-S subgroups, while recovered after 1 month in SPVI-R and CPVI-R subgroups. *P < 0.05.

There was no difference in preablation HRV parameters between successfully ablated patients and those with recurrence after either ablation technique. The HRV parameters SDNN, SDANN, rMSSD, PNN50, LF, HF, and LF/HF decreased significantly after SPVI or CPVI (Figures 1 and 2). Moreover, SDNN and rMSSD were significantly lower in patients without recurrence than in those with recurrence after either ablation technique (SPVI-S versus SPVI-R: SDNN 91.8 ± 32.6 versus 111.5 ± 36.2 ms, rMSSD 47.4 ± 32.3 versus 55.2 ± 35.2 ms; CPVI-S versus CPVI-R: SDNN 83.0 ± 35.6 versus 101.0 ± 40.7 ms, rMSSD 41.1 ± 22.9 versus 59.2 ± 44.8 ms; all *P* < 0.05). Additionally, there were no differences in postablation HRV parameters between the SPVI-S and CPVI-S subgroups or between the SPVI-R and CPVI-R subgroups (Figure 3). Serial changes during the entire followup indicated that SDNN remained attenuated during 12-month followup in SPVI-S and CPVI-S subgroups, whereas in SPVI-R and CPVI-R subgroups, it recovered to pre-ablation levels after 6 months. Reduction of rMSSD was observed during 12-month followup in SPVI-S and CPVI-S subgroups, while recovered shortly after 1 month in both SPVI-R and CPVI-R subgroups (Figure 4).

### 3.3. Predictors of AF Recurrence

**Figure 5:**
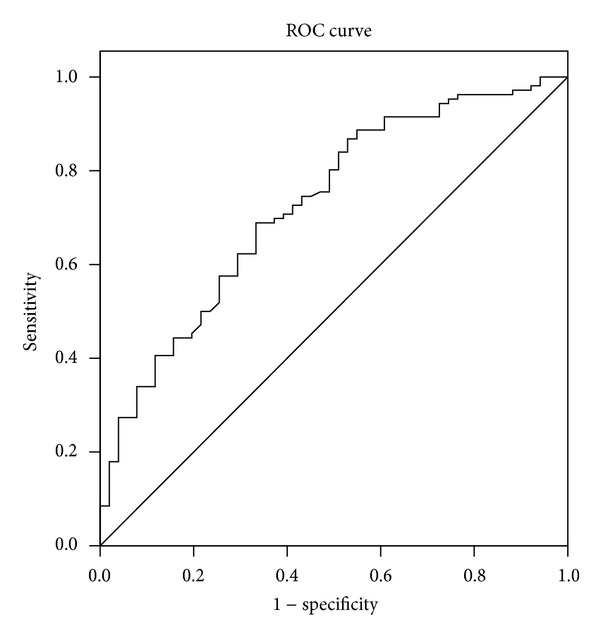
Receiver operator characteristic (ROC) curve indicated the predictive value of SDNN for long-term success of AF ablation. Area under the ROC curve was 0.71. The cut-off value was 91.9, with a sensitivity and specificity of 68.9% and 66.7%.

SDNN was identified as the only predictor of AF recurrence by multivariate logistic regression analysis (OR 1.015, 95% CI 1.005–1.025; *P* = 0.002). A receiver operator characteristic (ROC) curve analysis was performed to determine the value of postablation SDNN for prediction of AF recurrence (Figure 5). The area under the ROC curve was 0.71. Postablation SDNN <91.9 was predictive of long-term success with a sensitivity and specificity of 68.9% and 66.7%, respectively, and the positive and negative predictive values for long-term success were 77.3% and 50.1%, respectively.

## 4. Discussion

### 4.1. Main Findings

The main findings of this present study are as follows. (1) All studied HRV parameters decreased significantly after SPVI or CPVI procedures, whether they were recurrent or not. (2) In both SPVI and CPVI groups, the SDNN and rMSSD were significantly lower in successfully ablated patients than in recurrent patients. (3) As shown by multivariate logistic regression analysis, SDNN was the only independent predictor of PAF recurrence after different ablation therapies.

### 4.2. Both SPVI and CPVI Induce Denervation

HRV is regarded as an indicator of the dynamic interaction and balance between the sympathetic and parasympathetic nervous systems. Among time-domain and frequency-domain parameters, rMSSD, PNN50, and HF have been assumed to reflect parasympathetic nervous activity, whereas SDANN and the LF/HF ratios have been considered to reflect sympathetic nervous activity. SDNN and LF generally mirror overall vagosympathetic modulation. Previous studies demonstrated an immediate decrease of HRV after either SPVI or CPVI [6, 8, 10]. Consistent with these studies, we also found that SDNN, SDANN, rMSSD, PNN50, LF, HF, and LF/HF markedly decreased after SPVI or CPVI procedures. 

To understand the relationship between denervation and PVI, a review of the complex network of autonomic nerves in the atrium and PVs is necessary. Through pathological examination, Armour et al. [11] identified 5 atrial ganglionated plexi (GP) as follows: superior right atrial GP, superior left atrial GP, posterior right atrial GP, posteromedial left atrial GP, and posterolateral left atrial GP. Similarly, by means of high-frequency stimulation, Po et al. [12] found four major left GPs as follows: superior left GP, anterior right GP, inferior left GP and inferior right GP. Vaitkevicius et al. [13] examined the intrinsic neural structures of 35 intact LA-PV complexes stained transmurally for acetylcholinesterase using a stereomicroscope. Abundant extensions of epicardial nerves penetrate the PV walls transmurally and form the subendothelial neural network beneath the roots of 4 PVs. All parasympathetic nerves ultimately synapse on GP, whereas sympathetic fibers may directly innervate the myocardium or synapse on intrinsic cardiac ganglia [14]. Tan et al. [15] reported that adrenergic and cholinergic nerves both have higher densities in the LA region within 5 mm of the PV-LA junction than in regions further (>5 mm) away from the junction or in the PVs. 

In clinical practice, SPVI targets preferential electrical connections between PVs and LA, whereas CPVI targets the PV antrum with linear ablation encircling the ipsilateral PVs 0.5–1.0 cm outside of the PV ostia. Therefore, SPVI and CPVI could both result in damage to GPs and induce denervation. Because parasympathetic and sympathetic nerves are highly colocalized in tissues and cells [15], both components involved were attenuated after ablation. 

### 4.3. Effect of Denervation on Long-Term Efficiency of AF Ablation

The effects of autonomic modulation of cardiac electrophysiological properties have been well established. Experimental and clinical studies have demonstrated that both sympathetic and parasympathetic components are involved in the pathogenesis of AF [16]. Increased vagal tone is associated with a shortened atrial effective refractory period and increased dispersion [3, 17], whereas sympathetic stimulation may lead to afterdepolarization and triggered firing [18], which facilitate the initiation and maintenance of AF. Therefore, denervation effects may have potential therapeutic significance for AF, and various studies have established a link between autonomic denervation and short-term success after ablation.

Pappone et al. [5] first proposed that denervation effects may contribute to the prevention of AF recurrence. At 12 months of followup, PAF patients free of AF recurrence were characterized by more pronounced HRV changes. Yamada et al. [6] compared PAF recurrences after either SPVI or CPVI at 1 year of followup. Vagal modification was equally created during both PVI procedures. Comparisons of HRV in the immediate aftermath of ablation revealed lower levels of rMSSD, HF, and LF/HF in patients with no AF recurrence than in those with recurrence. Multivariate analysis revealed that HF and LF/HF were independent predictors of PAF recurrence. Yamaguchi et al. [19] performed box isolation on AF patients, which is a procedure characterized by the complete isolation of four PVs and a larger LA area compared with routine CPVI. After a followup of 16 ± 5 months, significant HRV attenuation was associated with a greater suppression of AF recurrence. Onorati et al. [20] observed that fat pad ablation during the Maze procedure significantly decreased AF recurrence at discharge and after 12.8 months of followup. Recent research from our laboratory also demonstrated that isolation of the right upper pulmonary vein attenuated vagal innervation of the atria and suppressed AF inducibility [21]. Ablation of the epicardial fat pad could effectively prevent acute atrial electrical remodeling and AF [17]. Ablation of the complex fractionated atrial electrogram (CFAE) also attenuates vagal modulation of the atria, thereby suppressing AF mediated by enhanced vagal activity [22]. 

However, there were also experiments with inconsistent results. Hirose et al. [23] found that partial right atrial vagal denervation had facilitated rather than prevented initiation of vagally mediated AF in mongrel dogs. After ablation, atrial electrophysiological properties indicated increased dispersion of refractoriness and nonuniform slow conduction. Oh et al. [24] argued that fat pad ablation only resulted in short-term denervation. AF inducibility was strongly augmented by vagal stimulation 4 weeks after FP ablation. Therefore, it remains controversial whether denervation provides long-term benefits for AF ablation. 

In the present study, we performed a five-year followup. After either PVI technique, patients with no PAF recurrence had significantly lower levels of SDNN and rMSSD than those with PAF recurrence. A logistic analysis confirmed that only SDNN, which represents an overall reflection of autonomic modulation, was predictive of PAF recurrence. Previous reports largely emphasized the effects of vagal denervation on AF ablation. Given that the vast majority (>95%) of ganglion cells are cholinergic but over 90% of ganglia also contain adrenergic innervations [15], the complex network of both components renders the selective elimination of either type during AF ablation almost impossible. The present study indicates that overall vagal and sympathetic denervation by either procedure may contribute to the long-term efficiency of AF ablation. Therefore, we speculated that more pronounced vagosympathetic denervation effects after ablation are crucial for a better outcome after AF ablation. Beyond PVI, denervation may be another common mechanism underlying different procedures for PAF. 

Possible mechanisms could be that suppression of autonomic nerves may alter atrial electrophysiological properties. Studies have revealed that vagally released acetylcholine (ACh) can induce Ca^2+^ overload and facilitate activation of the atrial ACh-regulated potassium current (*I*_K,ACh_), which results in abbreviation of the ERP and AF inducibility. Norepinephrine released from sympathetic nerve endings enhances the Ca^2+^ transient, and persistent Ca^2+^ transient elevation in phase 3 of the action potential may activate the Na^+^-Ca^2+^ exchange current and induce late phase 3 early afterdepolarization. Late phase 3 early afterdepolarization initiates focal discharge and AF [25, 26]. Therefore, denervation induced by either ablation could suppress the *I*_K,ACh_, Ca^2+^ overload, and the Na^+^-Ca^2+^ exchange current, which may lead to subsequent prolongation of atrial ERP and protection against AF.

## 5. Study Limitations

There were several study limitations that should be noted. First, complete elimination of vagal reflexes was not required in the present study. We demonstrated that CPVI induced more vagal reflexes than SPVI, but the incidence of vagal reflexes was not related to the long-term outcome of AF ablation. One possible explanation may be that we administered local anesthesia rather than general anesthesia, which could lead to vagal responses because of pain during the procedure. Secondly, we did not use implantable loop recorders or remote monitoring during followup, so the success rate for AF ablation may be overestimated because of asymptomatic AF episodes. Finally, previous studies have demonstrated LAD to be a predictor of AF recurrence after ablation, whereas in our study, there was no difference concerning the LAD between successful or recurrent subgroups. One possible explanation is that patients involved in our study had a relatively shorter AF history and less pronounced left atrial remodeling. 

## 6. Conclusions

In conclusion, both SPVI and CPVI induce immediate autonomic denervation after ablation. Denervation may contribute to the long-term success of PAF ablation. Beyond PVI, denervation may be a common mechanism underlying different ablation strategies for PAF.

## Figures and Tables

**Table 1:** Baseline and procedural characteristics of subgroups.

	SPVI group			CPVI group			SPVI versus CPVI groups
	SPVI-S (*n* = 43)	SPVI-R (*n* = 21)	*P* value	CPVI-S (*n* = 76)	CPVI-R (*n* = 35)	*P* value	*P* value
Sex, M/F	25/18	15/6	NS	56/20	28/7	NS	NS
Age (year)	57.4 ± 11.7	58.5 ± 12.0	NS	60.3 ± 10.1	58.1 ± 9.2	NS	NS
AF history prior to PVI (month)	68.1 ± 76.0	67.0 ± 58.7	NS	55.8 ± 59.7	81.2 ± 80.7	NS	NS
LAD (mm)	35.9 ± 4.2	38.3 ± 4.7	NS	37.2 ± 3.5	36.2 ± 4.3	NS	NS
LVEF (%)	58.3 ± 2.8	59.0 ± 2.7	NS	58.1 ± 2.4	58.6 ± 2.2	NS	NS
HT (%)	34.9% (15/43)	47.6% (10/21)	NS	38.2% (29/76)	38.2% (13/35)	NS	NS
CHD (%)	4.6% (2/43)	9.5% (2/21)	NS	6.6% (5/76)	8.6% (3/35)	NS	NS
Ablation time (min)	47.5 ± 14.6	40.2 ± 12.9	NS	70.1 ± 25.4	68.5 ± 25.2	NS	*P* < 0.01
Vagal reflexes	4.6% (2/43)	9.5% (2/21)	NS	14.5% (11/76)	17.1% (6/35)	NS	*P* < 0.01

SPVI: segmental pulmonary vein isolation; CPVI: circumferential pulmonary vein isolation; LAD: left atrial diameter; LVEF: left ventricular ejection fraction; HT: hypertension; CHD: coronary heart disease. NS: not significant.
